# The nuclear transcription factor RelB functions as an oncogene in human lung adenocarcinoma SPC-A1 cells

**DOI:** 10.1186/s12935-018-0580-5

**Published:** 2018-06-26

**Authors:** Hualong Qin, Jun Zhou, Jingjing Xu, Li Cheng, Zaixiang Tang, Haitao Ma, Feng Guo

**Affiliations:** 1 Department of Thoracic Surgery, The First Affiliated Hospital of Soochow University, Suzhou, China; 2 Center for Clinical Laboratory, The First Affiliated Hospital of Soochow University, Suzhou, China; 3 Department of Biostatistics, Medical College of Soochow University, Suzhou, China; 4 Department of Oncology, Nanjing Medical University Affiliated Suzhou Hospital, Suzhou, 215001 China

**Keywords:** RelB, Non-small cell lung cancer, Proliferation, Migration, Irradiation

## Abstract

**Background:**

Lung cancer is a leading public health issue worldwide. Although therapeutic approaches have improved drastically in the last decades, the prognosis of lung cancer patients remains suboptimal. The canonical nuclear transcription factor kappa B (NF-κB) signalling pathway is critical in the carcinogenesis of lung cancer. The non-canonical NF-κB signalling pathway (represented by RelB) has attracted increasing attention in the pathogenesis of haematological and epithelial malignancies. However, the function of RelB in non-small cell lung cancer (NSCLC) is still unclear. Recently, high expression of RelB has been detected in NSCLC tissues. We have also demonstrated that RelB expression is an independent prognostic factor in NSCLC patients.

**Methods:**

The mRNA and protein expression of RelB in NSCLC tissues were detected by qRT-PCR and IHC assay. The cell growth of SPC-A1 cells was detected in real-time using the x-Celligence system and xenograft tumour assays. The proliferation capability of cells was detected using a CFSE assay. Cell apoptosis was measured using Annexin V/PI staining, cell cycle was analyzed by the cytometry. Cell migration abilities were detected using the x-Celligence system and wound healing assays. The relative amounts of the active and inactive gelatinases MMP-2 and MMP-9 were examined using gelatin zymography experiments. Apoptosis of RelB depletion SPC-A1 cells after ionizing radiation at 8 Gy. The expression of cellular proliferation signal pathway related-proteins were examined by Western blot analysis.

**Results:**

The expression of RelB increases in NSCLC tissues. High RelB expression was significantly correlated with advanced-metastatic stage in patients with NSCLC. RelB-silencing inhibits cell growth in vitro and in vivo. We found that RelB affected cell proliferation by regulating AKT phosphorylation. RelB silencing attenuates the migration and invasion abilities of SPC-A1 cells and is likely related to the down regulation of MMP-9 activity and Integrin β-1 expression. In addition, RelB modulated radiation-induced survival of NSCLC cells predominantly by regulating Bcl-xL expression.

**Conclusions:**

Given the involvement of RelB in cell proliferation, migration, invasion, and radio-resistance, RelB functions as an oncogene in NSCLC cells. Our data here shed light on unexplored aspects of RelB in NSCLC.

## Background

Lung cancer is a major public health issue in most countries [1]. Lung cancer is one of the most common malignancies and the leading cause of cancer-related death in China [2]. There are two main histological types of lung cancer: small-cell lung carcinoma (SCLC) and non-small-cell lung carcinoma (NSCLC). NSCLC accounts for more than 85% of all lung cancer. Due to histological diversity, NSCLC is sub-classified into adenocarcinomas and squamous cell carcinomas. Several driver genes, such as *EGFR*, *c*-*MET*, and the *ALK*-*EML4* fusion gene, have been thoroughly investigated and contribute to aberrant cell proliferation and apoptosis in NSCLC [3]. Diverse drugs that target these driver genes have been developed and are routinely used for NSCLC treatment [4]. However, it remains necessary to discover and understand molecular biomarkers involved in NSCLC progression.

The NF-κB family consists of NF-κB1 (p50 and its precursor p105), NF-κB2 (p52 and its precursor p100), RelA, RelB, and c-Rel [5]. There are two major NF-κB pathways, the canonical and non-canonical, represented by the RelA/p50 and RelB/p52 heterodimers, respectively. The NF-κB pathways play a crucial role in various biological processes, such as inflammation, immune response, cell proliferation, apoptosis, and B cell differentiation [6, 7]. Dysregulated NF-κB activation leads to aberrant cell proliferation and promotes metastasis, which contributes to the carcinogenesis of human cancers including NSCLC [8].

The majority of previous studies have analysed the biological mechanisms of canonical NF-κB activity in diverse cancers. Constitutive activation of NF-κB can promote cell proliferation and increase the metastatic potential of several malignancies. The constitutive expression of NF-κB is also indicative of decreased survival in certain solid tumours [8, 9].

The involvement of the non-canonical NF-κB pathway has been increasingly studied for the pathogenesis of different tumours. RelB is the main subunit in the non-canonical NF-κB pathway. In chronic lymphocytic leukaemia (CLL), RelB activity, together with RelA activity, functions importantly to maintain the basal survival of CLL cells. Low RelB activity is linked to a favourable prognosis for CLL patients [10]. Higher RelB expression has been demonstrated in oestrogen receptor α (ERα)-negative breast cancers, due in part to repression of RelB synthesis by ERα signalling [11]. Moreover, RelB activation is inversely associated with ERα-positive breast cancer patients and is indicative of unfavourable survival odds. Myoglobin is a possible surrogate marker of non-canonical NF-κB pathway activation in ERα-positive breast cancers [12]. In prostate cancer, RelB is highly expressed in androgen-independent prostate cancer cells and is correlated with a more aggressive phenotype [13].

In NSCLC, the function of canonical NF-κB activity has been extensively addressed [14, 15]. RelA functions importantly in K-Ras-induced lung cancer transformation. One upstream molecule of canonical NF-κB signalling, IKKβ, is a potential therapeutic target for K-Ras-induced lung cancer. Lung cancer cells lacking RelA are prone to undergo apoptosis [16]. Myeloid cell RelA is necessary to link smoke-induced inflammation with lung cancer growth and functions in the activation of Wnt/β-catenin signalling in murine and human tumour cells [17]. Generally, RelA activity plays a tumour-supportive role and functions as an independent prognostic factor in NSCLC.

Few studies have reported the function of non-canonical NF-κB activity in NSCLC. The cytoplasmic expression of RelB correlates with tumour stage, and the nuclear expression of RelB detected by immunohistochemistry (IHC) in tissue samples from NSCLC patients differs between tumours and non-neoplastic tissues [18]. The expression levels of RelA, RelB, and p50 are higher compared with that of p52/p100 in NSCLC. Importantly, RelB expression correlates with proliferating NSCLC cells and is an independent predictor of lymph node metastasis [19]. Recently, our studies have shown that RelB expression is present in lung adenocarcinoma and squamous cell carcinoma at different levels. NSCLC patients with high RelB expression have significantly shorter overall survival (OS) than those with low RelB expression [20]. Taken together, our previous findings suggest that RelB plays an important role in the carcinogenesis of NSCLC. However, the mechanism of RelB involvement in NSCLC remains unclear.

In this study, we systematically examined the biological significance of RelB in an adenocarcinoma cell line and NSCLC tissues. We observed that RelB interfered with many aspects of SPC-A1 cell behaviours, such as cell growth, migration and invasion, and radio-sensitivity. Taken together, these results reveal a tumour-supportive role of RelB in NSCLC.

## Methods

### Patient samples

A total of 130 NSCLC samples were obtained after informed consent from patients from the thoracic department of the First Affiliated Hospital of Soochow University who fulfilled the diagnostic and immune-phenotypic criteria for NSCLC. Use of patient samples and clinical data in this study was approved by the Clinical Research Ethics Committee of the hospital. Tumour tissue and adjacent non-neoplastic tissues were obtained after surgical treatment. The clinical-pathological characteristics of the patients are shown in Table 1.

**Table 1:** Relationship between RelB expression and clinicopathological characteristics

Characteristics	RelB expression		*p* value
	High n = 69 (53.1%)	Low N = 61 (46.9%)	
Gender			0.650
Male	31 (55.3)	25 (44.7)	
Female	38 (51.3)	36 (48.7)	
Age			0.590
Mean ± SD	60.56 ± 10.15	60.15 ± 10.31	
Range	33–83	38–87	
Smoking			0.603
Yes	23 (50.0)	23 (50.0)	
No	46 (54.8)	38 (45.2)	
Histology			0.948
ADC	49 (53.2)	43 (46.8)	
SCC	20 (52.6)	18 (47.7)	
Degree of differentiation			0.116
Low	26 (66.7)	13 (33.3)	
Middle	38 (48.1)	41 (51.9)	
High	5 (41.7)	7 (58.3)	
Depth of tumor invasion			0.010^a^
T1	15 (36.6)	26 (63.4)	
T2	31 (52.5)	28 (47.5)	
T3	14 (73.7)	5 (26.3)	
T4	9 (81.8)	2 (18.2)	
Lymph node metastasis			0.048^a^
N0	24 (44.4)	30 (55.6)	
N1	35 (54.7)	29 (45.3)	
N2	10 (83.3)	2 (16.7)	
Distant metastases			0.006^a^
No	56 (48.7)	59 (51.3)	
Yes	13 (86.7)	2 (13.3)	
TNM stage			< 0.001^a^
I	13 (33.3)	26 (66.7)	
II	21 (42.9)	28 (57.1)	
III	22 (81.5)	5 (18.5)	
IV	13 (86.7)	2 (13.3)	

*ADC* adenocarcinoma, *SCC* squamous cell carcinoma, *NSCLC* non-small cell lung cancer

^a^ The difference had statistically significance

### Cell culture and transfection

The human lung adenocarcinoma cancer cell line SPC-A1 was purchased from the Shanghai Institute for Biological Sciences (Shanghai, China). A shRNA carrying a sequence targeting the *RelB* gene (5′-GCACAGATG AATTGGAG-AT-3′) was subcloned into the pSilencer3.1-H1-neo plasmid (Thermo Scientific™, China). The recombinant pSilencer3.1-psRelB and the scrambled control plasmids were then transfected into SPC-A1 cells using Lipofectamine 2000 (Thermo Scientific™, China) according to the manufacturer’s instruction. Cell clones were selected using G418.

#### Western blot analysis

Cells (10 × 10^6^) were lysed in RIPA buffer according to standard instructions to obtain whole-cell extracts. Protein concentration was determined using a DC protein assay kit (Bio-Rad, USA). The whole-cell proteins were denatured and fractionated using SDS-PAGE. After electrophoresis, the proteins were transferred to nitrocellulose membranes. Membranes were probed with different antibodies (Abs), washed, and incubated with appropriate secondary Abs. Proteins were detected and scanned with an Odyssey system (LI-COR Biosciences, USA). β-actin Ab was used as an internal control. RelA (sc-372), RelB (sc-226), c-Rel (sc-70), p105/50 (sc-7178), and p100/52 (sc-3017) Abs were purchased from Santa Cruz Biotechnology (Shanghai, China). The Phospho-AKT Pathway Antibody Sampler Kit (9916) and the Integrin β-1 (9699), Bcl-2 (sc-7382), and Bcl-xl (2764) Abs were purchased from Cell Signaling Technology (Shanghai, China). Actin (AO1215a) Ab was purchased from Abgent (Suzhou, China).

### Quantitative real-time PCR (qRT-PCR)

Total RNA was isolated using TRIzol reagent (Tiangen Biotech Co., Ltd., Beijing, China) and then quantified by a NanoDrop 1000. Two microgram of RNA was reverse-transcribed to cDNA and amplified using 2×LC480 SYBR-green IMaster Mix (Roche) with a LightCycler 480 instrument (Roche Diagnostics, China). Primers were designed and synthesized by Invitrogen Corporation (China). For data analysis, target gene transcripts were quantified in comparison to *β*-*actin* as a reference.

#### Cell growth assays

Cell growth was monitored using a x-Celligence RTCA instrument (Roche) according to the manufacturer’s instructions. The impedance of SPC-A1 cell growth was continuously monitored for 96 h, and the ‘cell index’ value was measured, which indicates the number of cells seeded. Cells were seeded in wells at a density of 10,000 cells/well. The data was collected and analysed by RTCA software 1.2.

### Cell proliferation assays

Cells (1 × 10^6^) suspended in phosphate-buffered saline (PBS) were stained with CFSE (Molecular Probes) at 37 °C for 10 min. Then, pre-cooled RPMI-1640 media without serum was added to the cells to stop the staining, and the cells were subsequently washed three times with RPMI-1640 media. Finally, cells were cultured for 24, 48 and 72 h and harvested at the indicated times to detect fluorescence intensity of the stain using a FACS Calibur™ cytometer.

### Cell apoptosis and cell cycle analysis

For apoptosis assays, cells were stained at the indicated times with Annexin V and propidium iodide (PI) according to the manufacturer’s instructions (Invitrogen, China). For cell cycle assays, cells cultured for 48 h were harvested and fixed with 70% ethanol overnight at 4 °C. Subsequently, single cell suspensions were prepared to stain DNA using PI. Cell apoptosis and cell cycle status were measured with a FACS Calibur™ cytometer (BD Biosciences).

### Cell migration assays

For cell migration assays, cells suspended in 100 μl FBS-free RPMI-1640 media were added into the upper chamber of a CIM-plate (40,000 cells/well). RPMI-1640 (170 μl) containing 10% FBS was added to the lower chamber of each well. Following cell attachment, cell migration towards the lower chamber was continuously monitored using an x-Celligence RTCA instrument.

### Scratch healing assays

Cells were scratched using a 200 μl pipette tip, washed three times with PBS, and then cultured with RPMI-1640. Wound closure was observed at 0, 24, 48, 72 and 96 h, with a light System Microscope IX71.

### Gelatinase zymography

Cells at a confluence of 80% were washed twice with PBS and were changed to 2 ml RPMI-1640 media without serum for further culture. After 48 h, culture media was harvested and filtered through 0.45 μm filters. Samples were loaded and fractioned on an 8% SDS-PAGE gel supplemented with 0.1% gelatin under non-reducing conditions. Then, the gels were washed twice for 30 min with 2.5% Triton X-100. The gels were incubated at 37 °C overnight in substrate buffer, stained with 0.5% Coomassie Blue R250 for 30 min, and destained. Finally, gelatinases were used to digest the gelatin to produce clear bands during enzyme renaturation.

### Radiation exposure

Each type of cells received a single dose of 8 Gy at 2.25 Gy/min via a 6 MeV linear accelerator (Simens Primus-M), RT. The distance between the radiation source and the cells was 100 cm.

### IHC

Formalin-fixed, paraffin-embedded (FFPE) sections were dewaxed in xylene and rehydrated with graded ethanol. Then, the FFPE tissue sections were pretreated with 0.01 M citrate buffer (pH 6.0) followed by treatment with 3% hydrogen peroxide (H_2_O_2_) to block endogenous peroxidase. After washing three times with PBS (pH 7.4), the sections were incubated with anti-RelB antibody overnight at 4 °C. The sections were then incubated with biotinylated goat anti-rabbit IgG. Finally, 3,3-diaminobenzine was used to visualize the immunoreactive products. The results were evaluated using a System Microscope IX71.

### Xenograft tumour assays

Four-week-old male BALB/c mice were purchased from Shanghai Experimental Animal Corporation (China). All the animal experiments in this study comply with the animal rights, the national guidelines of animal experiments management and the ethical principles. Then, 5 × 10^6^ cells were resuspended in PBS and injected into the right oxter of a mouse. Ten mice were used for each experimental group. One group of mice was injected with SPC-A1-shctrl cells and another with SPC-A1-shRelB cells. Mice were housed under sterile conditions during all experiments and were sacrificed after 3 weeks. The weight and dimension of tumours borne by the mice were measured. The tumours were then fixed in formalin and embedded in paraffin for subsequent histological analyses.

### Statistical analysis

All experiments were performed at least three times. The data are presented as the mean ± standard deviation (SD) from experiments in replicate. All statistical analysis was performed using GraphPad software. The differences between groups were evaluated by the Student’s *t* test, and *p *< 0.05 was defined as a statistically significant difference.

## Results

### The expression of RelB increases in NSCLC tissues

**Fig. 1:**
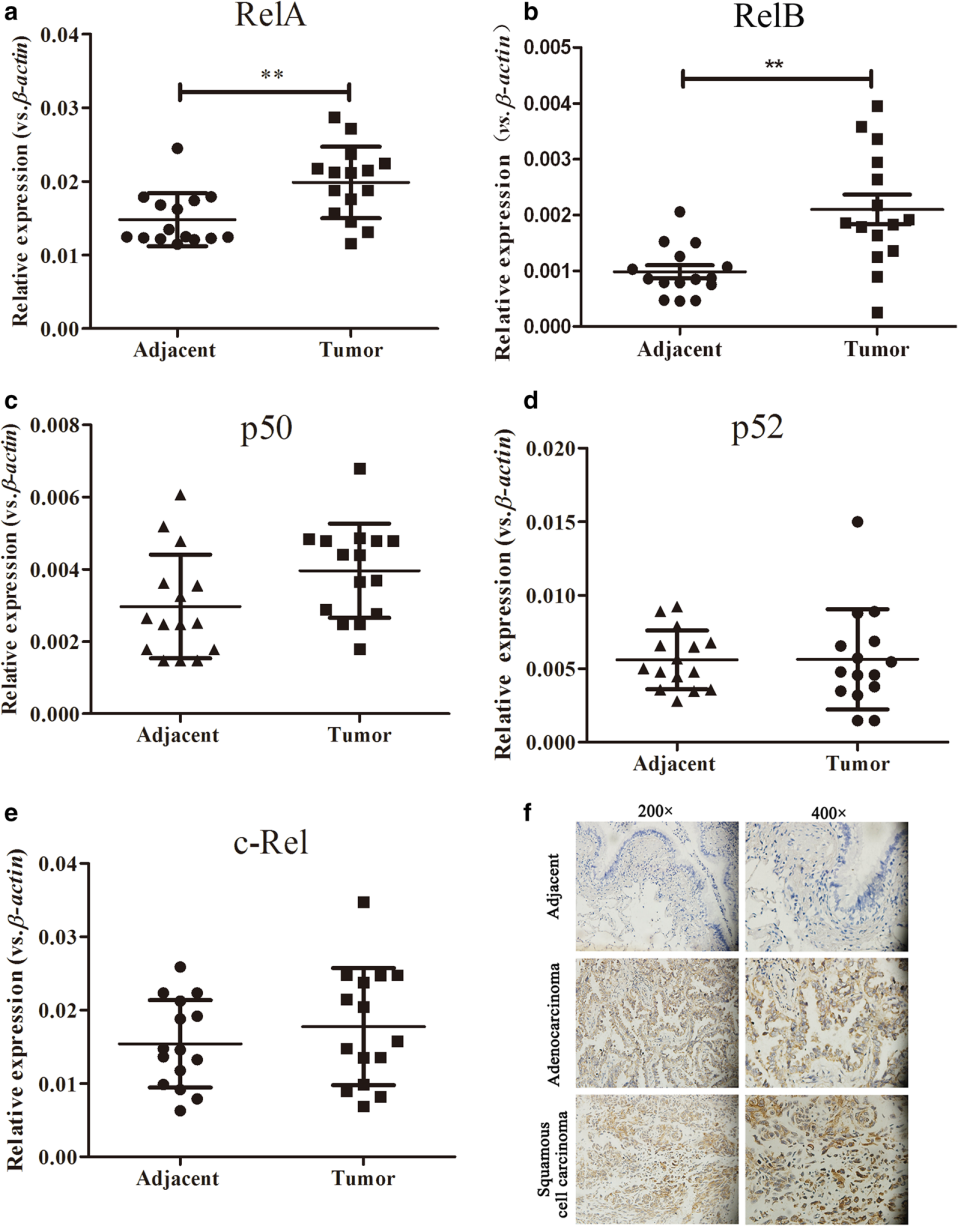
RelB expression in human non-small cell lung cancer and adjacent non-neoplastic tissues. **a**–**e** mRNA expression of NF-κB subunits in tumours and adjacent non-neoplastic tissues. Total RNA was extracted from adjacent non-neoplastic (N) or tumour (T) tissues, and mRNA expression of NF-κB subunits was quantified using qRT-PCR after normalization to *β*-*actin*. **f** Representative images of RelB expression using IHC staining. Adjacent non-neoplastic and tumour tissue images of adenocarcinomas and squamous cell carcinomas (×200). **p < 0.01

The average mRNA levels of the NF-κB subunits in 15 pairs of NSCLC or adjacent non-neoplastic tissues were detected by qRT-PCR. The mRNA expression levels of *RelA* and *RelB* in the NSCLC tissues were clearly higher than that of the adjacent non-neoplastic tissues (Fig. 1a, b), while the mRNA expression levels of *p50*, *p52*, and *cRel* in the NSCLC tissues were comparable to those of the adjacent non-neoplastic tissues (Fig. 1c–e). There was a statistically significant difference in the mRNA levels of *RelA* (*p *< 0.05) and *RelB* (*p *< 0.01) between the NSCLC and the adjacent non-neoplastic tissue.

IHC was performed to examine the expression of RelB at the protein level in 130 FFPE tissues from patients with NSCLC. The heterogeneity of RelB expression was observed in adenocarcinomas and squamous cell carcinomas. In adenocarcinoma tissues, the expression of RelB was detected in both the nucleus and the cytoplasmic portions of the tumour cells, while the expression of RelB was nearly undetectable in the adjacent non-neoplastic tissues (Fig. 1f). High RelB expression was detected in 53.3% (49/92) of the adenocarcinomas. Similarly, RelB was present in both the nucleus and the cytoplasmic portions of the squamous cell carcinomas (Fig. 1f). High RelB expression was detected in 52.6% (20/38) the squamous cell carcinomas. There was no statistically significant difference in the frequency of high RelB expression between the adenocarcinomas and the squamous cell carcinomas (*p *= 0.948). The association between RelB expression and the clinical characteristics of the NSCLC patients were further analysed. High RelB expression was significantly correlated with depth of tumour invasion (*p *= 0.010), lymph node metastasis (*p *= 0.048), distant metastases (*p *= 0.006), and TNM stage (*p *< 0.001) in patients with NSCLC (Table 1).

### Establishing a shRNA-RelB cell line

**Fig. 2:**
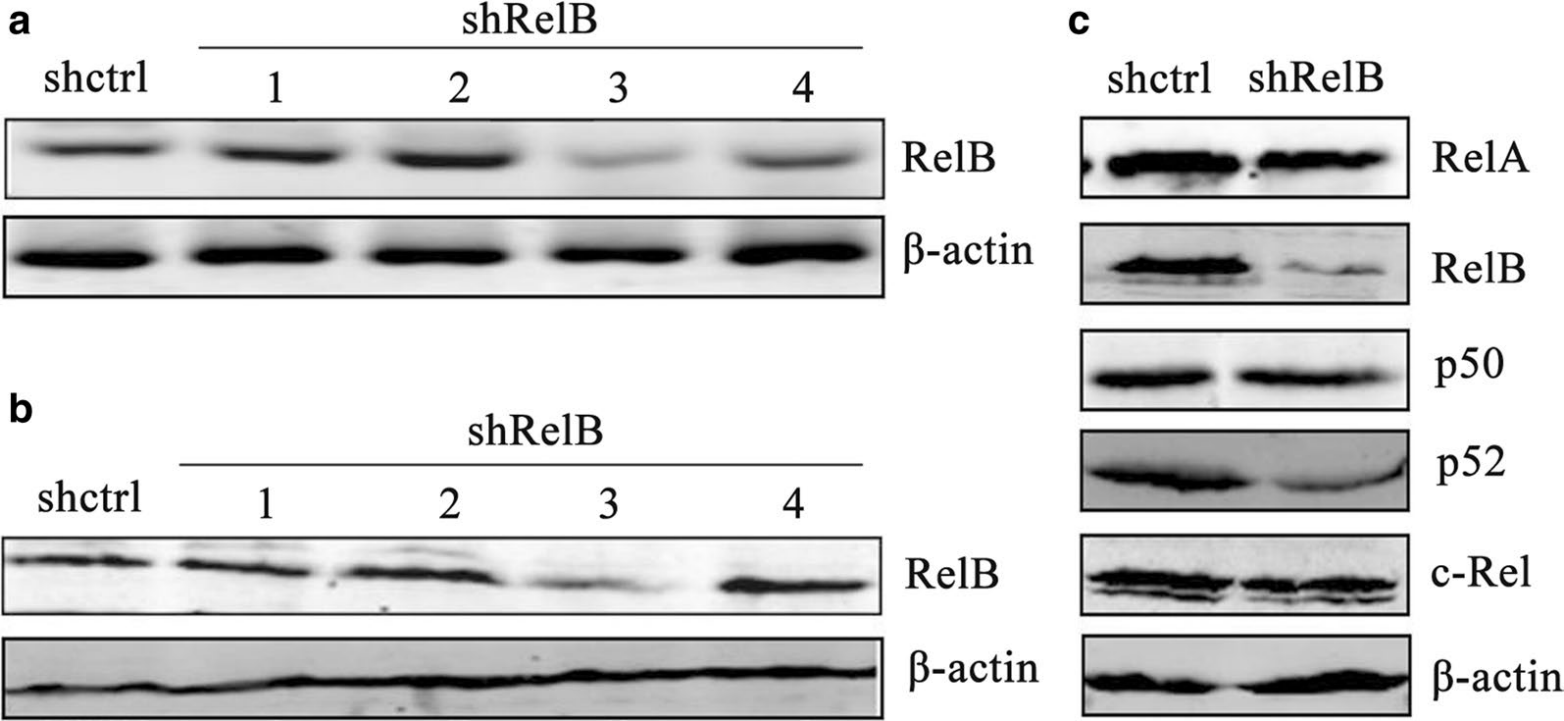
Establishment of a SPC-A1 cell line expressing low levels of RelB. **a** *RelB* mRNA expression of the established cell lines as determined by PCR analysis and normalized to *β*-*actin* gene expression. **b** Protein levels of RelB expression in the SPC-A1-shctrl and SPC-A1-shRelB cell lines were determined by Western blotting. The level of each protein was normalized against β-actin. **c** RelB silencing affects the expression of other NF-κB subunits. Western blot analysis of individual NF-κB family members protein expression. Protein expression in the whole portion was normalized against β-actin

Plasmid-based RelB shRNA or control shRNA was stably transfected into SPC-A1 cells. The shRNA-RelB and shRNA-control cells were selected for in the presence of G418 (300 ng/μl). The selected monoclones were further examined for RelB expression by RT-PCR and Western blotting. As shown in Fig. 2a and b, both *RelB* mRNA and RelB protein expression levels were markedly decreased in clone No. 3, indicating successful RNA interference (RNAi) with the *RelB* gene. Western blotting was carried out to investigate whether RelB silencing affected the expression of other NF-κB subunits. As shown in Fig. 2c, RelB silencing by RNAi did not affect the expression levels of the canonical NF-κB members RelA, p50, and cRel. The expression level of the non-canonical NF-κB member p52 was slightly decreased when the *RelB* gene was silenced in the SPC-A1-shRelB cells.

### RelB-silencing inhibits cell growth in vitro and in vivo

**Fig. 3:**
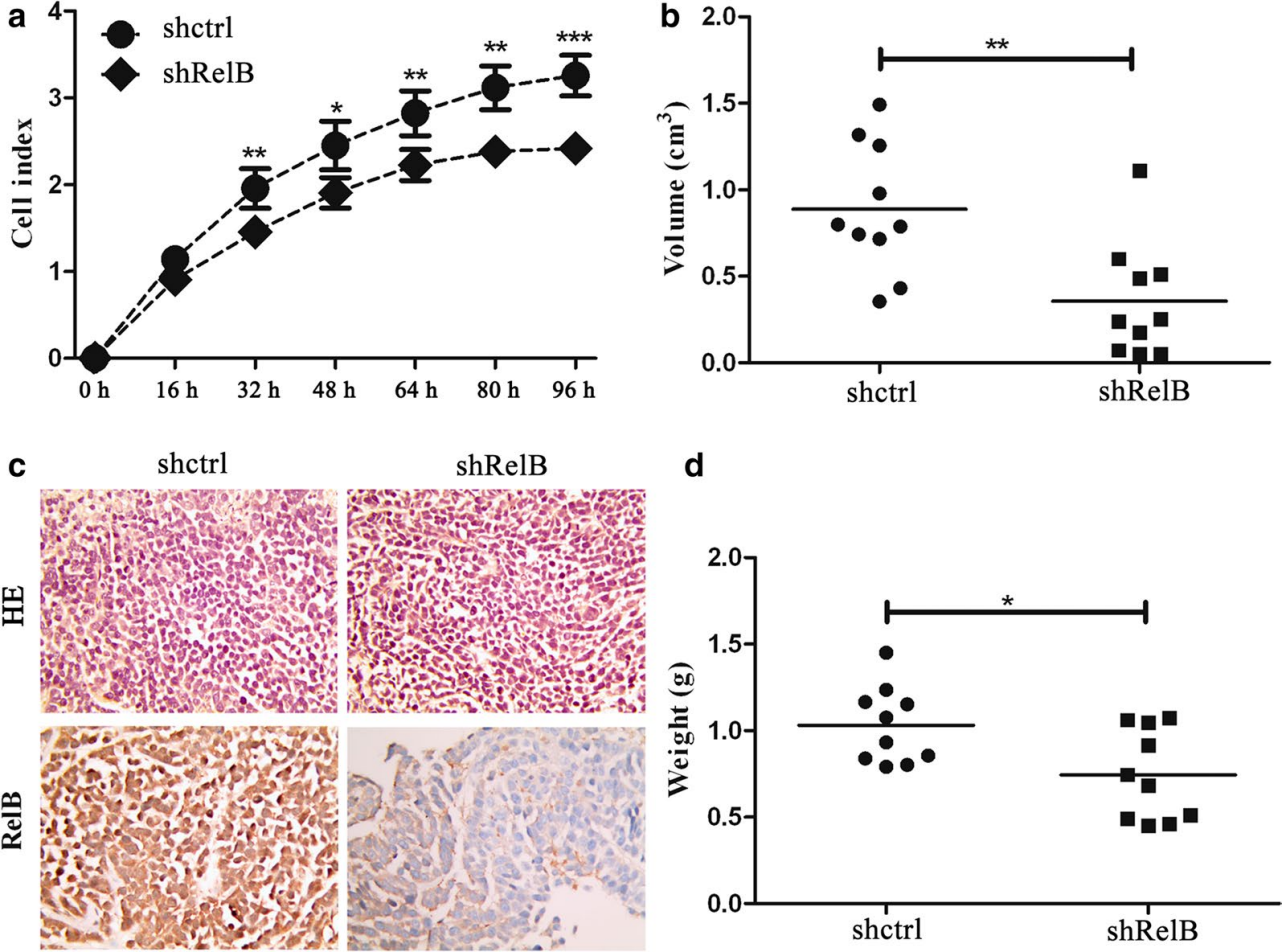
RelB silencing inhibits growth of SPC-A1 cells *in vivo* and in vitro. **a** The cell growth curves of SPC-A1-shctrl and SPC-A1-shRelB cells were monitored continuously for 96 h using a x-Celligence system. Each well was plated with 10,000 cells. **b** and **d** The volume and weight of tumours formed subcutaneously from the SPC-A1-shRelB cells and SPC-A1-shctrl cells, respectively. **c** Representative images of RelB expression of tumours formed subcutaneously from the SPC-A1-shRelB cells or SPC-A1-shctrl cells, using HE staining. Original magnification, ×200. *p < 0.05; **p < 0.01; ***p < 0.001

The growth of SPC-A1 cells was detected in real-time using the x-Celligence system and E-plates. As shown in Fig. 3a, the SPC-A1-shRelB cells grew much slower the SPC-A1-shctrl cells. There was a statistically significant difference in the growth of SPC-A1-shRelB cells and SPC-A1-shctrl cells during the 32- to 96-h continuously monitored time period. The cell growth curve clearly indicated that RelB silencing had a suppressive effect on SPC-A1 cell growth in vitro (Fig. 3a).

To further investigate the role of RelB silencing in SPC-A1 cell growth in vivo, SPC-A1-shRelB cells or SPC-A1-shctrl cells were injected subcutaneously into nude mice. Three weeks post-injection, the average volume of tumours formed subcutaneously from the SPC-A1-shRelB cells or SPC-A1-shctrl cells was (0.36 ± 0.31) cm^3^ and (0.89 ± 0.37) cm^3^, respectively (Fig. 3b). The average weight of tumours formed subcutaneously from the SPC-A1-shRelB cells and SPC-A1-shctrl cells was (0.74 ± 0.26) g and (1.03 ± 0.22) g, respectively (Fig. 3d). There were significant differences in the volume (*p *= 0.003) and weight (*p *= 0.046) of subcutaneous tumours derived from the two established cell lines.

Histological analyses of tumours formed were carried out to confirm the expression of RelB in the xenografts. IHC showed that RelB could be detected in the tissues injected with SPC-A1-shctrl cells. However, RelB was nearly undetectable in the tissues injected with SPC-A1-shRelB cells (Fig. 3c). Taken together, these data indicate that RelB silencing in SPC-A1 cells suppresses cell growth in vitro and in vivo.

### RelB-silencing inhibits cellular proliferation ability

**Fig. 4:**
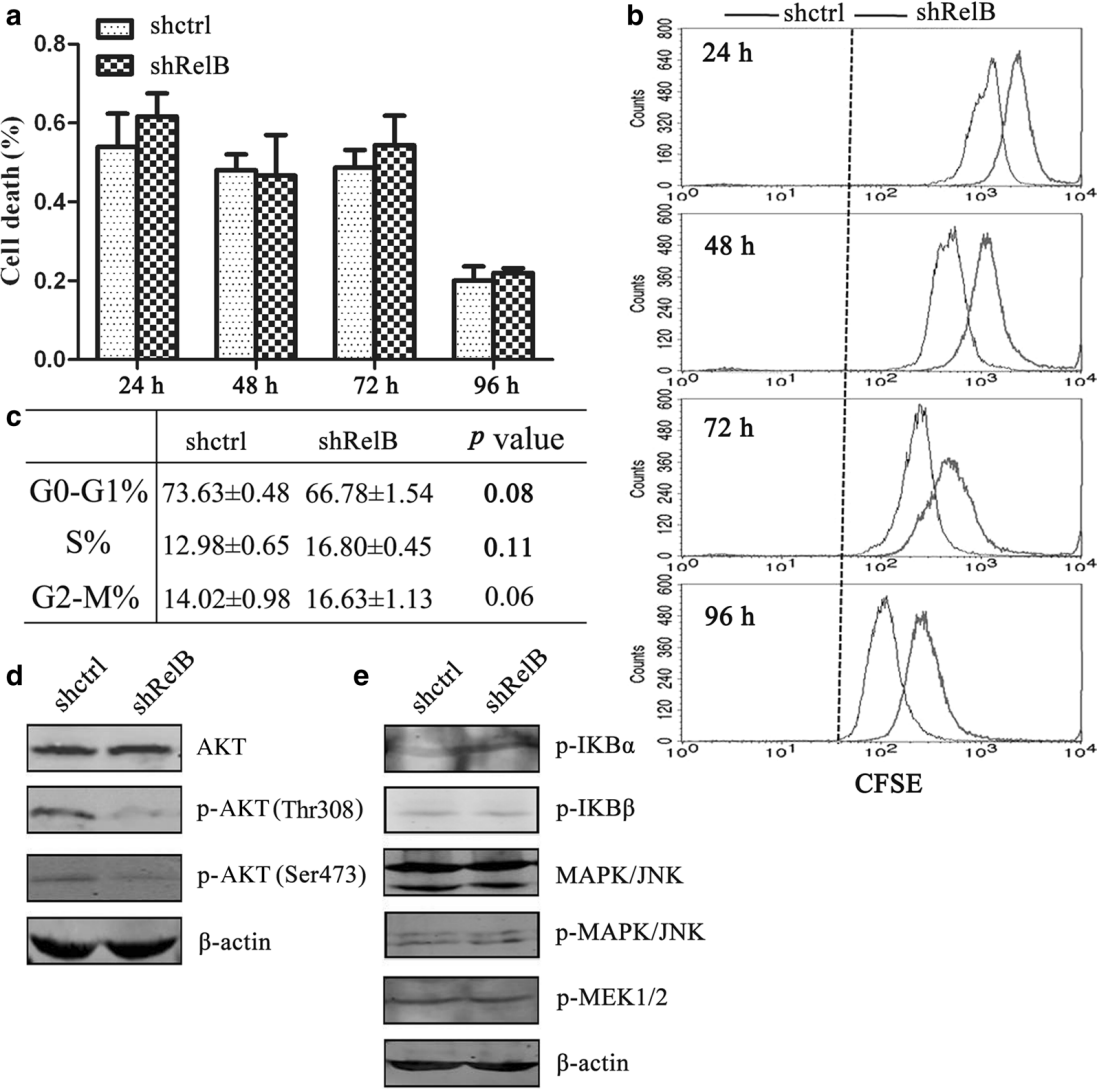
RelB silencing inhibits proliferation of SPC-A1 cells. **a** The bar chart represents the percentage of apoptotic cells within the two established cell lines. **b** CFSE cell proliferation assays were performed using flow cytometry at 24, 48, 72 and 96 h. **c** Cell cycle transitions between the two established cell lines were determined by flow cytometry. Data representative of the three phases (G0–G1, S and G2-M) are displayed in the table. **d**, **e** Western blot analysis of the expression of cellular proliferation signal pathway related-proteins. Protein expression levels were normalized against β-actin

To further investigate the mechanism underlying suppression of SPC-A1 cell growth as a result of RelB silencing, cellular apoptosis and proliferation assays were performed. Annexin V/PI assays were carried out to quantitatively analyse cellular apoptosis. Both established cell lines underwent spontaneous apoptosis in a time-dependent manner. However, no statistically significant difference in the spontaneous apoptosis rate was found between the SPC-A1-shRelB cells and the SPC-A1-shctrl cells at different time points (Fig. 4a). The proliferation capability of cells was detected using a CFSE assay. As shown in Fig. 4b, the fluorescence intensity of CFSE was attenuated in both established cell lines in a time-dependent manner. However, the SPC-A1-shRelB cells proliferated at a markedly slower rate than the SPC-A1-shctrl cells during the 24- to 96-h continuously monitored time period (Fig. 4b). Thus, RelB silencing decreased the proliferation of SPC-A1 cells. Cell cycle assays were performed using flow cytometry. The percentages of SPC-A1-shRelB cells in the three cell cycle phases (G0–G1, S, and G2-M) were 66.78 ± 1.54, 16.80 ± 0.45 and 16.63 ± 1.13%, while those of the SPC-A1-shctrl cells were 73.63 ± 0.48, 12.98 ± 0.65 and 14.02 ± 0.98%, respectively. There were no statistically significant differences in cell cycle progression between the two groups (Fig. 4c).

To gain insights into the mechanisms of RelB-silencing attenuation of SPC-A1 cell proliferation, cellular proliferation signal pathway related-proteins were examined by Western blot analysis. As shown in Fig. 4d, expression of total AKT was detected in both cell lines, while phosphorylated AKT protein expression (phosphorylation sites at Thr 308 and Ser 473) of SPC-A1-shRelB cells was distinctly reduced compared to SPC-A1-shctrl cells. However, the protein levels of p-MEK1/2, JNK1/2, p-JNK1/2, and p-IκBα/β were comparable between the shctrl and shRelB SPC-A1 cells (Fig. 4e). Taken together, these results suggest that the AKT signalling pathway is inactivated by RelB silencing in SPC-A1 cells, which diminishes cellular proliferation. Therefore, it is likely that RelB plays a pivotal role in growth of SPC-A1 cells due to its regulation of cellular proliferation.

### RelB silencing attenuates cell migration and invasion abilities

**Fig. 5:**
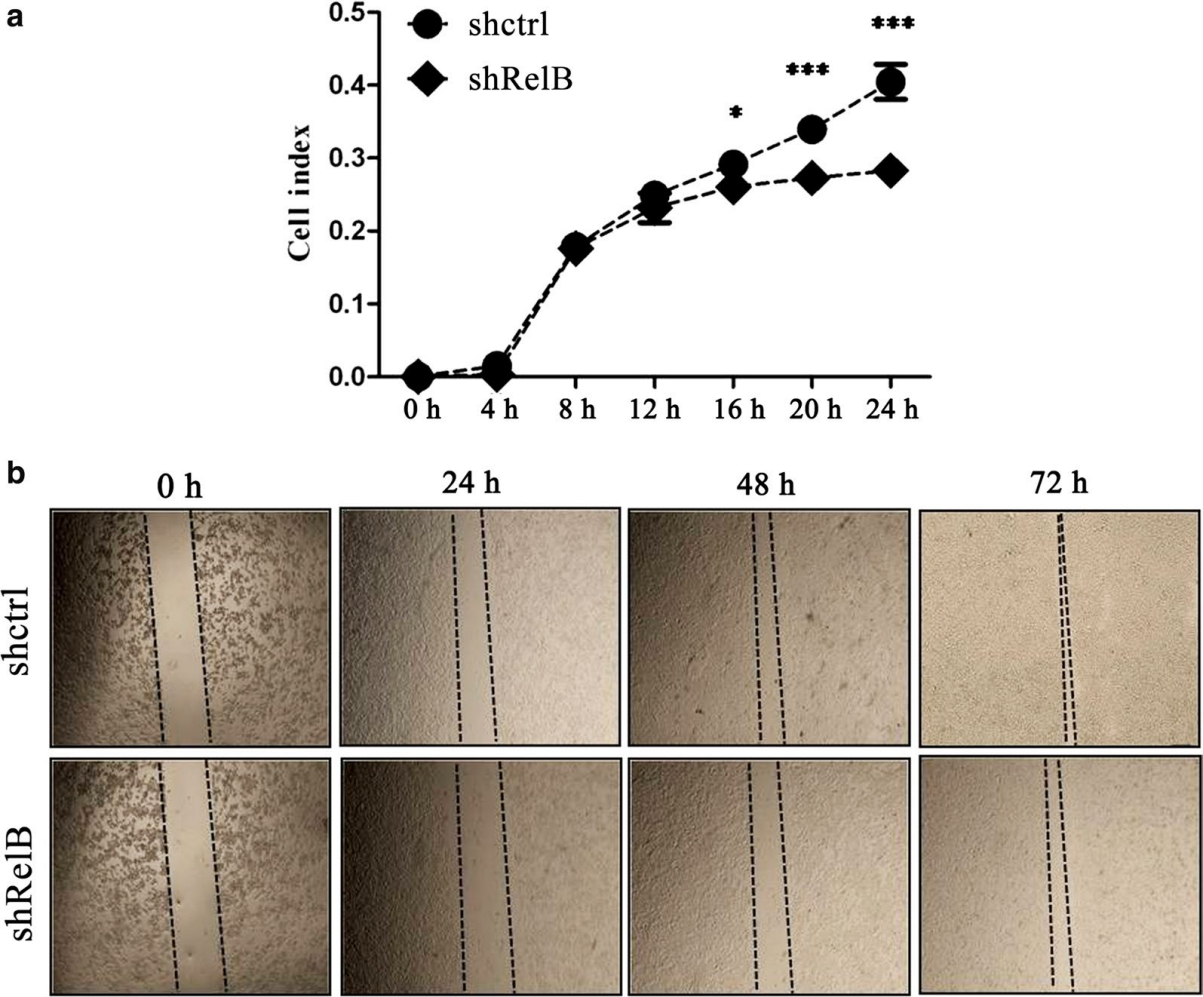
RelB silencing hampers the migration of SPC-A1 cells. **a** The migration ability of SPC-A1-shctrl and SPC-A1-shRelB cells was monitored continuously for 24 h, using a x-Celligence system. Each well was plated with 40,000 cells. **b** Analysis of the migration ability of the two established cell lines was detected by wound healing assays at 0, 24, 48, and 72 h. *p < 0.05; **p < 0.01; ***p < 0.001

To assess whether RelB might affect the migration ability of SPC-A1 cells, cell migration assays were performed in real-time using a x-Celligence system. As shown in Fig. 5a, the migration curves for SPC-A1-shctrl cells and SPC-A1-shRelB cells begin to separate at the 8 h-time point. The SPC-A1-shRelB cells migrated at a distinctly slower rate than the SPC-A1-shctrl cells. There were significant differences in the migration abilities of the two established cell lines during the 16–24 h time period.

Wound healing assays were also carried out to evaluate whether RelB affects the migration ability of SPC-A1 cells. A scratched cell monolayer was generated, and images were captured after culturing the cells for 72 h. After 72 h, the SPC-A1-shRelB cells migrated from the edge of the scratch toward the scratch centre at a much slower rate than the SPC-A1-shctrl cells (Fig. 5b).

**Fig. 6:**
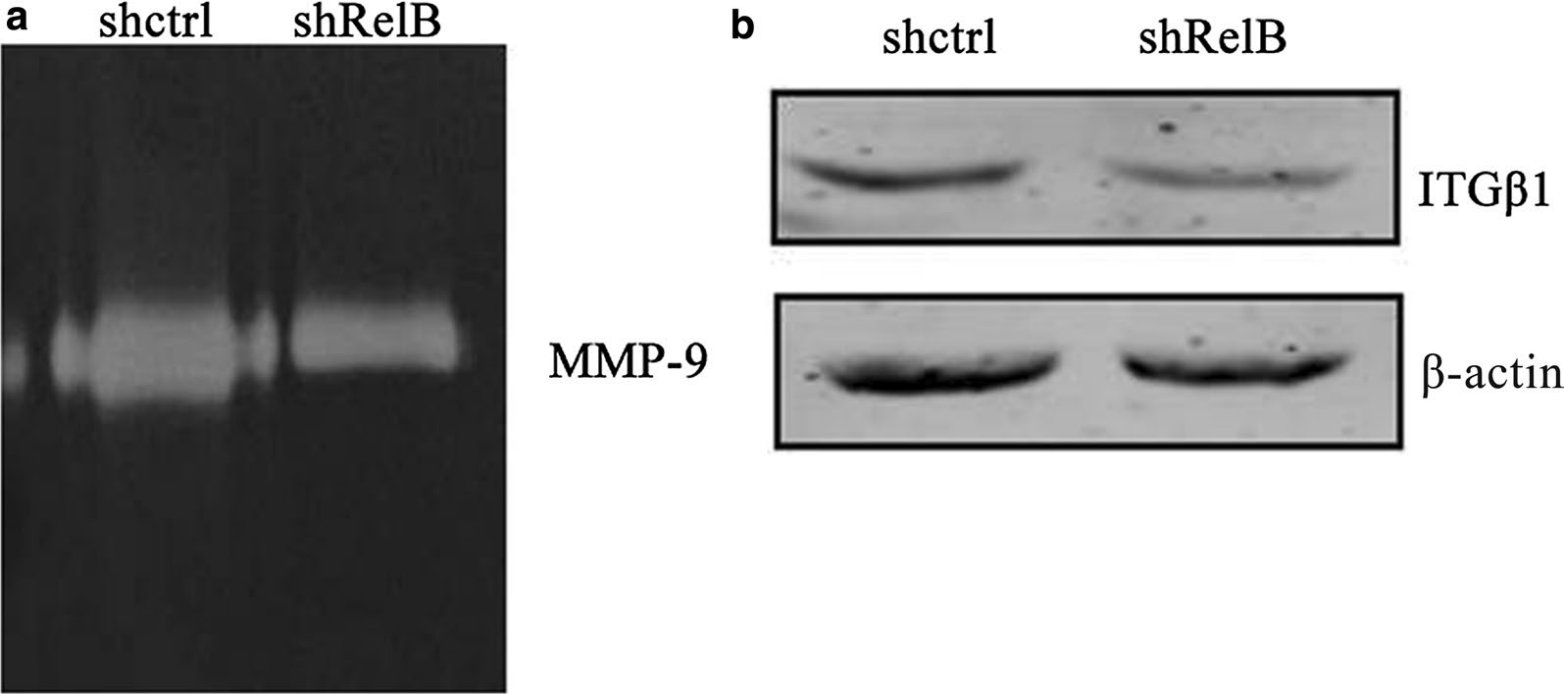
RelB silencing attenuates the invasion ability of SPC-A1 cells. **a** Gelatin zymography experiments to test the activity of MMP-2 and MMP-9. **b** Western blot analysis of Integrin β-1 (ITGB1) protein level. β-actin expression is shown as a loading control

The relative amounts of the active and inactive gelatinases MMP-2 and MMP-9, major members of the matrix metalloproteinases family, were examined using gelatin zymography experiments. As shown in Fig. 6a, MMP-2 activity nearly undetectable while MMP-9 activity was inhibited by RelB silencing. The protein level of Integrin β-1 was decreased in the SPC-A1-shRelB cells compared to the SPC-A1-shctrl cells (Fig. 6b). Together, these results demonstrate that RelB silencing attenuates the migration and invasion abilities of SPC-A1 cells and is likely related to the down regulation of integrin β-1 expression.

### RelB silencing increases the radio-sensitivity of SPC-A1 cells

**Fig. 7:**
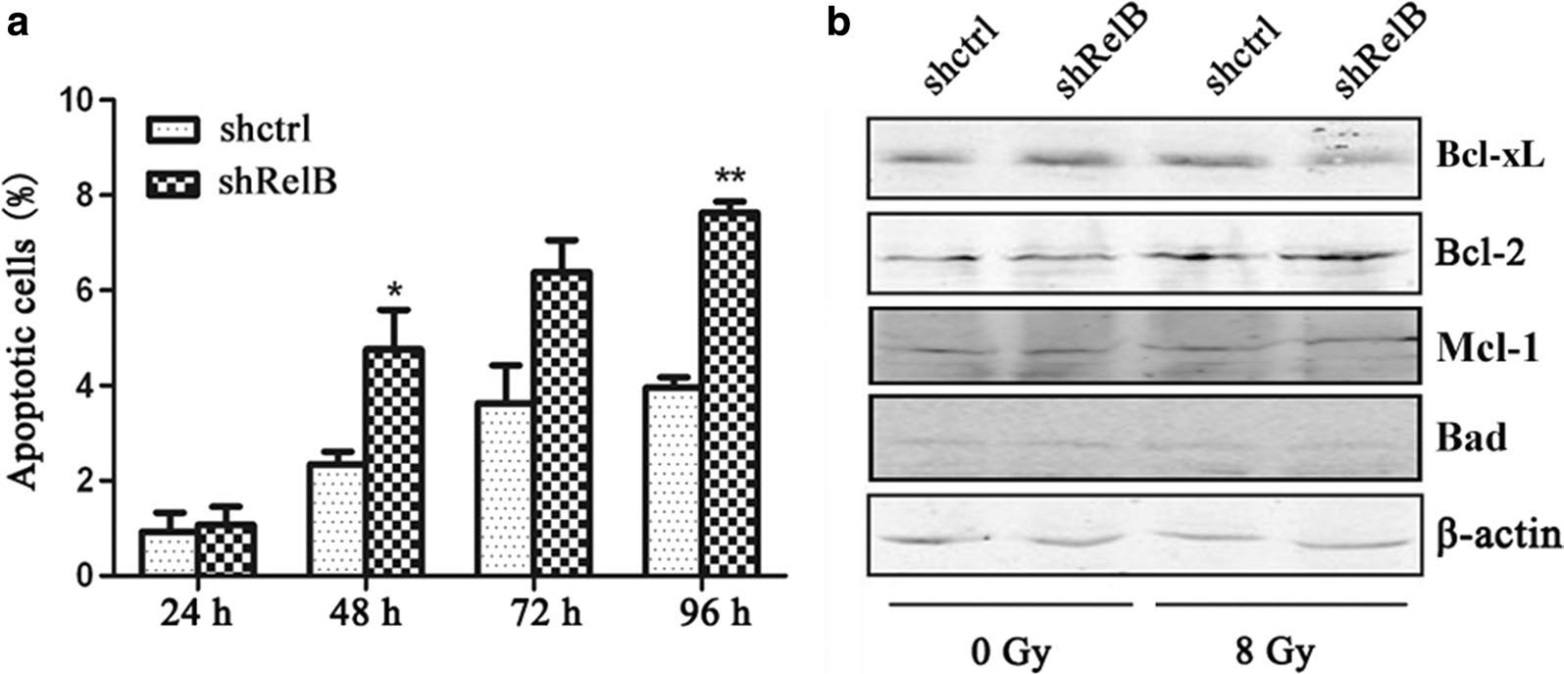
RelB silencing increases the radio-sensitivity of SPC-A1 cells. **a** Analysis of apoptotic cells at 24, 48, 72 and 96 h after 8 Gy of radiation exposure, as analysed by flow cytometry. **b** Western blot analysis of Bcl-2 and Bcl-xL protein levels at 96 h after 0 and 8 Gy of radiation exposure, normalized against β-actin. *p < 0.05; **p < 0.01

To investigate whether RelB affects the radio-sensitivity of SPC-A1 cells, SPC-A1-shRelB and SPC-A1-shctrl cells were subjected to ionizing radiation at 8 Gy. After radiation exposure, apoptosis was measured using Annexin V/PI staining at 24, 48, 72, and 96 h. As shown in Fig. 7a, the apoptosis frequency was increased in both established cell lines in a time-dependent manner. The apoptosis rates of the SPC-A1-shRelB cells were much higher than those of the SPC-A1-shctrl cells. There was a statistically significant difference in the apoptosis rate between the two groups at 48 and 96 h (*p *< 0.05 and *p *< 0.01, respectively). These results indicate that SPC-A1 cells lacking RelB expression were more sensitive to radiation-induced apoptosis compared to control cells.

The expression levels of Bcl-xL, Bcl-2, Mcl-1 and Bad were examined by Western blot analysis 96 h after radiation exposure. As shown in Fig. 7b, the expression of Bcl-xL protein was decreased in SPC-A1-shRelB cells compared to SPC-A1-shctrl cells 96 h after 8 Gy of radiation. The expression level of Bcl-2, Mcl-1 and Bad were unchanged after irradiation. Overall, these results indicate that RelB silencing in SPC-A1 cells enhances radio-sensitivity, likely because of the decreased expression of the Bcl-xL.

## Discussion

In this study, we systemically investigated the role of RelB in NSCLC. In primary NSCLC samples, RelB expression was increased in tumour tissue both at the mRNA and protein levels. In in vitro assays, we found that RelB silencing affects lung adenocarcinoma SPC-A1 cell proliferation, which can be attributed to inhibited AKT activity. Furthermore, RelB silencing significantly affected the migration and invasion abilities of SPC-A1 cells, likely due to inhibition of MMP-9 activity and integrin β-1 expression. In addition, we showed that RelB silencing increased the sensitivity of SPC-A1 cells to radiation by increasing radiation-induced apoptosis.

Previously, we analysed the expression of RelB in NSCLC tissues by IHC and studied its clinical significance. High RelB expression is correlated with the TNM stage of NSCLC and is significantly associated with shorted OS in NSCLC patients. For the first time, we also found that high RelB expression might be an independent prognostic factor in NSCLC [20].

In this study, we primarily focused on investigating the involvement of RelB function in NSCLC. In vitro, cell growth was significantly inhibited upon introduction of shRNA-RelB into SPC-A1 cells. Although cell apoptosis and cell cycle transition were not affected, cell proliferation was suppressed by RelB silencing, which contributed to reduced cell growth in vitro. In vivo, RelB silencing inhibited the volume and weight of subcutaneous tumours established by the subcutaneous xenograft model. Therefore, the in vivo data is consistent with the in vitro data for reduced cell growth in the context of RelB silencing. Recently, it was reported that increased RelB expression enhances EC cell growth by regulating cell cycle transition and cell proliferation, leading to endometrial cell tumourigenicity [21]. In SCID mice, RelB overexpression leads to a lag in the initiation of 22Rv1-induced tumours. RelB overexpression stimulates 22Rv1 cell proliferation and reduces colony formation in soft agar [22]. Our study is in line with these previous findings, which highlight a role for the alternative NF-κB pathway in cell proliferation and includes the AKT, PI3K, MEK1/2 and JNK1/2 signalling pathways. AKT, also known as protein kinase B (PKB), is a serine/threonine-specific protein kinase. The function of AKT is to regulate cell proliferation and cell survival by phosphorylating and activating or inactivating numerous downstream cytoplasmic and nuclear substrates [23]. Imatinib mesylate (Gleevec, STI571), a tyrosine kinase inhibitor, can enhance RelB nuclear translocation in androgen-responsive LNCaP prostate cancer cells. STI571 inhibits the phosphoinositide 3-kinase (PI3K)–AKT–IKKα pathway in PC-3 cells by decreasing the phosphorylation levels of PI3K and AKT (Ser 473) [24]. In our study, the suppressed phosphorylation of AKT (at both Ser 473 and Thr 308) contributed to the reduced cell proliferation ability of SPC-A1 cells in the absence of RelB expression. However, the protein levels of p-MEK1/2, JNK1/2, p-JNK1/2, and p-IκBα/β were comparable between shctrl and shRelB cells, indicating that the relationship between RelB and AKT signalling requires further investigation.

Previously, we found that high RelB activity together with RelA activity maintains the basal survival of CLL cells [10]. RelB is also a crucial positive regulator of cell survival in multiple myeloma (MM) [25, 26]. Loss of RelB expression also significantly attenuates cell survival in mesenchymal gliomas. High RelB expression strongly correlates with rapid tumour progression and poor patient survival rates [27]. We have reported the RelB silencing in the androgen-independent prostate cancer cell line DU145 significantly affects cell survival. Bcl-2 plays a critical role in regulating both spontaneous and radiation-induced apoptosis [28]. Usually, RelB functions as an oncogenic driver of tumour cell survival. However, unlike previous reports on other malignancies, RelB silencing did not interfere with the survival of lung adenocarcinoma SPC-A1 cells. However, constitutively activated RelA in SPC-A1 cells, in the presence of RelB knockdown, is indeed a powerful survival regulator.

Furthermore, we found that RelB silencing suppressed the migration and invasion abilities of SPC-A1 cells by cell migration assays and gelatin zymography experiments. MMP-9 activity was clearly inhibited by knockdown of RelB while MMP-2 activity was nearly undetectable. Very few studies have reported the effect of RelB function and its mechanism of action on the migration and invasion abilities of NSCLC cells. In our previous studies, we found that RelB silencing inhibits prostate cancer cell migration and invasion due to the reduction of Integrin β-1 expression [28]. For the first time, we have provided evidence that in vitro knockdown of RelB also suppresses the migration and invasion abilities of lung adenocarcinoma SPC-A1 cells. The findings here are correlated with the clinical analysis of RelB expression in NSCLC patients. High RelB expression was found in NSCLC patients in advanced stages of the disease with tumour invasion, lymph node metastasis, and distant metastases. Therefore, RelB does play a role in the metastasis of NSCLC.

Integrins are a family of heterodimeric transmembrane cell surface receptors responsible for cellular adhesion to extracellular matrix (ECM) protein. Integrin β-1, encoded by the *ITGB1* gene, is a key regulator of the switch from cellular dormancy to metastatic growth in vitro and in vivo. Integrin β-1 overexpression has been found in diverse epithelial malignancies during metastasis. In lung cancer, Integrin β-1 knockdown suppresses cell invasion and metastasis [29]. Overexpression of Integrin β-1 has been found in the human NSCLC cell line PC9/AB2, which exhibits a 576-fold decrease in gefitinib sensitivity compared to the parental PC9 cell line. In addition, the adhesion and migration abilities of PC9/AB2 cells are increased. Overexpression and activation of Integrin β-1 speeds the epithelial–mesenchymal transition (EMT) [30]. Blocking RelB expression prevents the induction of Integrin β-1 expression and interferes with the attachment ability of small cell lung cancer H69 cells [31]. Reduced expression of Integrin β-1 has been detected in DU145 cells lacking RelB expression, which is linked to the suppressed migration and invasion abilities of the cells [28]. In this study, a similar phenomenon was observed, indicating that RelB functions as an oncogene regulating metastasis in NSCLC cells. Since the *ITGB1* promoter does not contain the NF-κB consensus sequence, investigation of the regulation of Integrin β-1 expression by RelB is warranted.

Radio-resistance is a major problem encountered in the treatment of NSCLC patients. It is imperative to develop a strategy to overcome radio-resistance of NSCLC. Nevertheless, the molecular mechanisms underlying the radio-resistance of NSCLC cells remains poorly understood. Many molecules and microRNAs are involved in modulating radio-resistance in NSCLC cells [32]. A marked increase of radio-sensitivity in human NSCLC cell lines often occurs after inhibiting survivin expression with YM155 (sepantronium bromide), a specific survivin inhibitor [33]. RelB is a crucial factor in the differential radiosensitization effects of ascorbic acid in prostate cancer cells and normal prostate epithelial cells [34]. RelB silencing in RM-1 prostate cancer cells inhibits Bcl-xL expression and enhances radiosensitivity by regulating radiation-induced apoptosis [35, 36]. In our previous study, we showed that RelB silencing in DU145 cells enhances radio-sensitivity, which is mediated by inhibiting the expression of the *Bcl*-*2* gene [28]. Thus, RelB overexpression, which leads to high alternative NF-κB pathway activity, confers radio-resistance in prostate cancer cells. In our current study, we found that knockdown of RelB increased the apoptosis frequency of SPC-A1 cells after exposure to 8 Gy radiation, which is in line with our previous studies in prostate cancer cells. A key mechanism by which the non-canonical NF-κB pathway controls cell apoptosis is through induced transcription of several anti-apoptotic genes, including *Bcl*-*xL* and *Bcl*-*2* [28, 37]. Bcl-xL, which belongs to the Bcl-2 family, plays a critical role in tumour progression and development [38]. The expression level of Bcl-xL was reduced in the SPC-A1-siRelB cells in response to ionizing radiation while the expression level of Bcl-2, Mcl-1 and Bad remained unchanged. Bcl-xL, a factor involved in both chemo-resistance and radio-resistance, has also been demonstrated to be regulated by RelB in epithelial malignant cells including NSCLC cells. These data indicate that Bcl-xL is a potential target gene of RelB in NSCLC cells. Thus, radio-sensitivity was increased after RelB silencing in SPC-A1 cells, which can likely be attributed to the reduction of Bcl-xL. However, the exact mechanisms underlying the downregulation of Bcl-xL after irradiation of SPC-A1 cells demands further investigation.

## Conclusions

Overall, our study reveals the critical role of RelB in the carcinogenesis of NSCLC cells. RelB silencing inhibited SPC-A1 cell growth as evidenced by a decrease in cellular proliferation. RelB silencing also conferred less aggressive phenotypes on SPC-A1 cells by attenuating their migration and invasion abilities. RelB silencing enhanced the radio-sensitivity of SPC-A1 cells, likely by reducing Bcl-xL expression. Taken together, these results suggest that RelB plays an important role in the carcinogenesis of NSCLC. Blockage of the alternative NF-κB pathway via RelB silencing is a promising approach for NSCLC therapeutic intervention.

## Abbreviations

NF-κB: nuclear transcription factor kappa B
NSCLC: non-small cell lung cancer
CLL: chronic lymphocytic leukaemia
IHC: immunohistochemistry
OS: overall survival
qRT-PCR: quantitative real-time PCR
PBS: phosphate-buffered saline
PI: propidium iodide
FFPE: formalin-fixed, paraffin-embedded
SD: standard deviation
MM: multiple myeloma
ECM: extracellular matrix
EMT: epithelial–mesenchymal transition
